# A new, easily generated mouse model of diabetic kidney fibrosis

**DOI:** 10.1038/s41598-019-49012-4

**Published:** 2019-08-29

**Authors:** Xiaolin He, Tianzhou Zhang, Monica Tolosa, Santosh Kumar Goru, Xiaolan Chen, Paraish S. Misra, Lisa A. Robinson, Darren A. Yuen

**Affiliations:** 1 Keenan Research Centre for Biomedical Science, Li Ka Shing Knowledge Institute, St. Michael’s Hospital, Toronto, Ontario Canada; 2 Department of Respiratory and Critical Care Medicine, Beijing Shijitan Hospital, Capital Medical University, Beijing, People’s Republic of China; 3 Hospital for Sick Children, Toronto, Ontario Canada

**Keywords:** Diabetes complications, Chronic kidney disease, Interstitial disease, Renal fibrosis

## Abstract

Our understanding of diabetic kidney disease pathogenesis has been hampered by the lack of easily generated pre-clinical animal models that faithfully recapitulate critical features of human disease. While most standard animal models develop manifestations of early stage diabetic injury such as hyperfiltration and mesangial matrix expansion, only a select few develop key late stage features such as interstitial fibrosis and reduced glomerular filtration rate. An underlying theme in these late stage disease models has been the addition of renin-angiotensin system hyperactivation, an important contributor to human disease pathogenesis. Widespread use of these models has been limited, however, as they are either labour intensive to generate, or have been developed in the rat, preventing the use of the many powerful genetic tools developed for mice. Here we describe the Akita^+/−^ Ren^+/−^ mouse, a new, easily generated murine model of diabetic kidney disease that develops many features of late stage human injury, including not only hyperglycemia, hypertension, and albuminuria, but also reduced glomerular filtration rate, glomerulosclerosis, and interstitial fibrosis.

## Introduction

Diabetic kidney disease is the leading cause of kidney failure in the United States, and is associated with significant morbidity and mortality^1,2^. Despite several major advances over the last 20 years, our understanding of diabetic nephropathy pathogenesis remains limited. A major barrier preventing further advances has been the lack of pre-clinical animal models that faithfully recapitulate all stages of human diabetic kidney disease. Indeed, most rodent models of diabetic nephropathy develop only functional and structural features mimicking early human disease such as hyperfiltration and glomerular basement membrane thickening, without progression to a more advanced phenotype that reflects the majority of patients who come to clinical attention^3^.

A key feature of advanced human diabetic nephropathy that most rodent models lack is the development of significant interstitial fibrosis. Multiple reports have linked interstitial fibrosis to disease progression, as interstitial matrix volume correlates closely with outcomes^4^. Moreover, pathophysiologic studies have suggested an important role for interstitial fibrosis as a driver of disease progression, in part by obliterating peritubular capillaries and thus reducing tubular oxygen delivery^5–7^. This failure to mimic a key event in the pathogenesis of human diabetic nephropathy has hampered our understanding of advanced diabetic kidney disease. More importantly, it has also prevented the rational design of pharmacologic agents targeting this common and critical disease stage. Not surprisingly, few safe and effective treatments are available for patients with advanced diabetic nephropathy, and as such many patients with diabetes progress to kidney failure^2^.

Although our understanding of diabetic nephropathy pathogenesis remains limited, decades of research have provided some important insights into key events that drive disease progression. Systemic and intrarenal renin angiotensin system (RAS) hyperactivation, for example, are well-established hallmarks of diabetic kidney disease^8,9^. Consistent with its important role in disease pathogenesis, RAS activation is a critical fibrogenic stimulus^10,11^, with RAS inhibition attenuating renal fibrosis and progression of kidney dysfunction in both animals^9,12–14^ and humans^15–18^. More recently, multiple reports have identified the related transcription co-factors Yes-Associated Protein (YAP) and transcriptional coactivator with PDZ-binding motif (TAZ) as important drivers of fibrosis progression in the kidney and other organs^19–22^. When activated, YAP and TAZ localize to the nucleus of both epithelial and mesenchymal cells, where they drive the expression of extracellular matrix genes via interaction with other pro-fibrotic transcription factors^19,23^.

Here we describe an easily generated mouse model of diabetes with RAS activation that develops significant interstitial fibrosis in concert with fibroblast YAP/TAZ activation, as well as many other features of advanced human diabetic nephropathy (DN), including glomerulosclerosis, elevated blood pressure, albuminuria, and reduced glomerular filtration rate.

## Results

### Hypertensive, diabetic Akita^+/−^ Ren^+/−^ mice develop renal dysfunction

**Figure 1:**
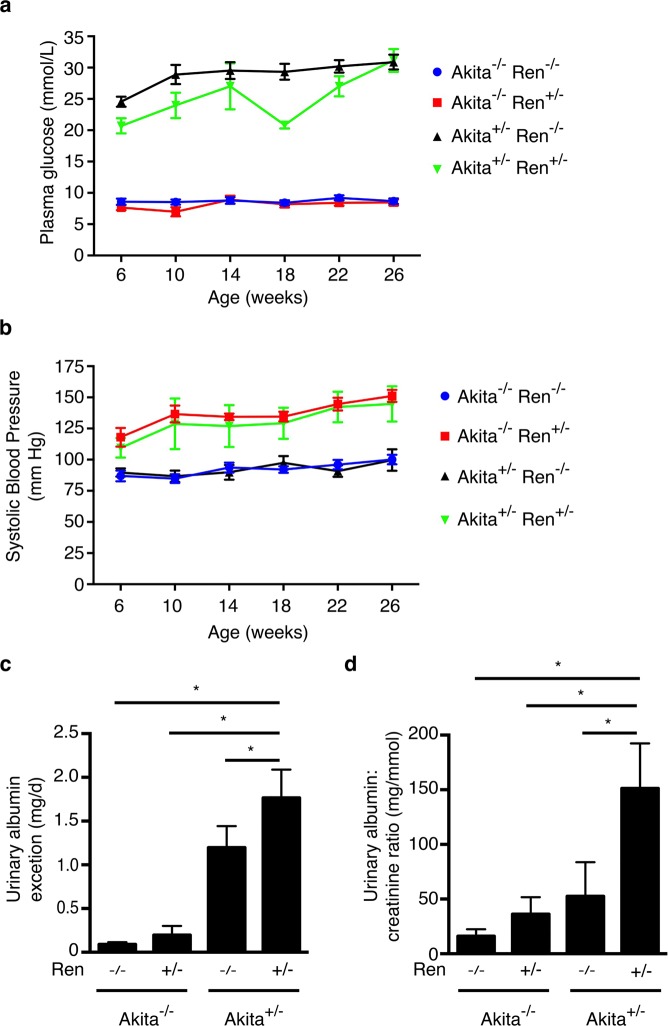
Akita^+/−^ Ren^+/−^ mice develop significant hyperglycemia, hypertension, and albuminuria over the course of 26 weeks. (**a**) Capillary blood glucose levels and (**b**) systolic blood pressure were measured monthly, beginning at 6 weeks of age. Urinary albumin excretion was measured just prior to sacrifice at 26 weeks of age as (**c**) daily urine albumin excretion rate and (**d**) a spot urinary albumin:creatinine ratio. A one-way ANOVA with a post-hoc Fisher’s least significant difference analysis was performed. *p < 0.05.

Mice for the study were generated by breeding male Akita^+/−^ with female Ren^+/−^ mice to produce Akita^−/−^ Ren^−/−^, Akita^−/−^ Ren^+/−^, Akita^+/−^ Ren^−/−^, and Akita^+/−^ Ren^+/−^ mice. Animals were followed for 26 weeks post-natally. As expected, the presence of the Akita mutation (Akita^+/−^ Ren^+/−^ and Akita^+/−^ Ren^−/−^ mice) resulted in hyperglycemia by 6 weeks of age that persisted until study end (Fig. 1a and Table 1). Similarly, the presence of the renin transgene (Akita^−/−^ Ren^+/−^ and Akita^+/−^ Ren^+/−^ mice) raised blood pressure, regardless of diabetes status (Fig. 1b and Table 1). However, compared to standard normotensive diabetic Akita^+/−^ Ren^−/−^ mice, the hypertensive diabetic Akita^+/−^ Ren^+/−^ mice developed significantly higher urine albumin excretion by study end (Fig. 1c,d). Consistent with their increased urinary albumin excretion, hypertensive diabetic Akita^+/−^ Ren^+/−^ mice also exhibited a significant loss of podocytes, a population of specialized epithelial cells that form an essential part of the glomerular filtration barrier (Supplemental Fig. 1).

**Table 1:** Functional and structural parameters of mice at study end.

n	Akita^−/−^ Ren^−/−^	Akita^−/−^ Ren^+/−^	Akita^+/−^ Ren^−/−^	Akita^+/−^ Ren^+/−^
	16	10	6	4
Phenotype	Non-diabetic, Normotensive	Non-diabetic, Hypertensive	Diabetic, Normotensive	Diabetic, Hypertensive
Plasma glucose (mmol/L)	9.1 ± 0.6	8.8 ± 0.7	30.2 ± 1.4^a,b^	29.0 ± 1.6^a,b^
HbA_1c_ (%)	5.1 ± 0.1	4.9 ± 0.1	13.4 ± 0.4^a,b^	13.0 ± 0.0^a,b^
Body weight (g)	44.2 ± 1.5	40.8 ± 1.1	35.8 ± 1.7a	34.2 ± 0.9^a,b^
Systolic blood pressure (mm Hg)	100 ± 4	151 ± 5a	99 ± 9^b^	145 ± 14^a,c^
Serum creatinine (μmol/L)	32.9 ± 2.3	35.8 ± 4.2	58.8 ± 4.8^a,b^	70.2 ± 3.1^a,b^
24 hour urine volume (mL)	2.2 ± 0.7	2.8 ± 0.8	51.0 ± 6.4^a,b^	33.2 ± 2.6^a,b,c^
Right kidney weight (μg)	245 ± 10	290 ± 20	492 ± 54^a,b^	445 ± 43^a,b^
Tibia length (mm)	19.9 ± 0.4	19.1 ± 0.6	18.5 ± 0.6	18.2 ± 0.6
Right kidney/tibia length (μg/mm)	12.3 ± 0.4	15.2 ± 1.0	27.0 ± 3.8^a,b^	24.4 ± 2.2^a,b^
Right kidney/body weight (μg/g)	5.6 ± 0.2	7.2 ± 0.6	13.7 ± 1.4^a,b^	13.0 ± 1.4^a,b^

^a^p < 0.05 vs. Akita^−/−^ Ren^−/−^. ^b^p < 0.05 vs. Akita^−/−^ Ren^+/−^. ^c^p < 0.05 vs. Akita^+/−^ Ren^−/−^. Data are presented as mean +/− standard error of the mean.

Akita^+/−^ Ren^+/−^ mice also exhibited significantly increased serum creatinine levels (a marker of reduced glomerular filtration rate) compared with both normotensive, non-diabetic Akita^−/−^ Ren^−/−^ mice and hypertensive, non-diabetic Akita^−/−^ Ren^+/−^ mice (Table 1). As has been described previously, normotensive, diabetic Akita^+/−^ Ren^−/−^ mice also experienced an increase in serum creatinine by 26 weeks of age^24^, although hypertensive, diabetic Akita^+/−^ Ren^+/−^ mice demonstrated a trend towards even higher serum creatinine levels (Table 1).

### Akita^+/−^ Ren^+/−^ mice develop significant fibrosis

**Figure 2:**
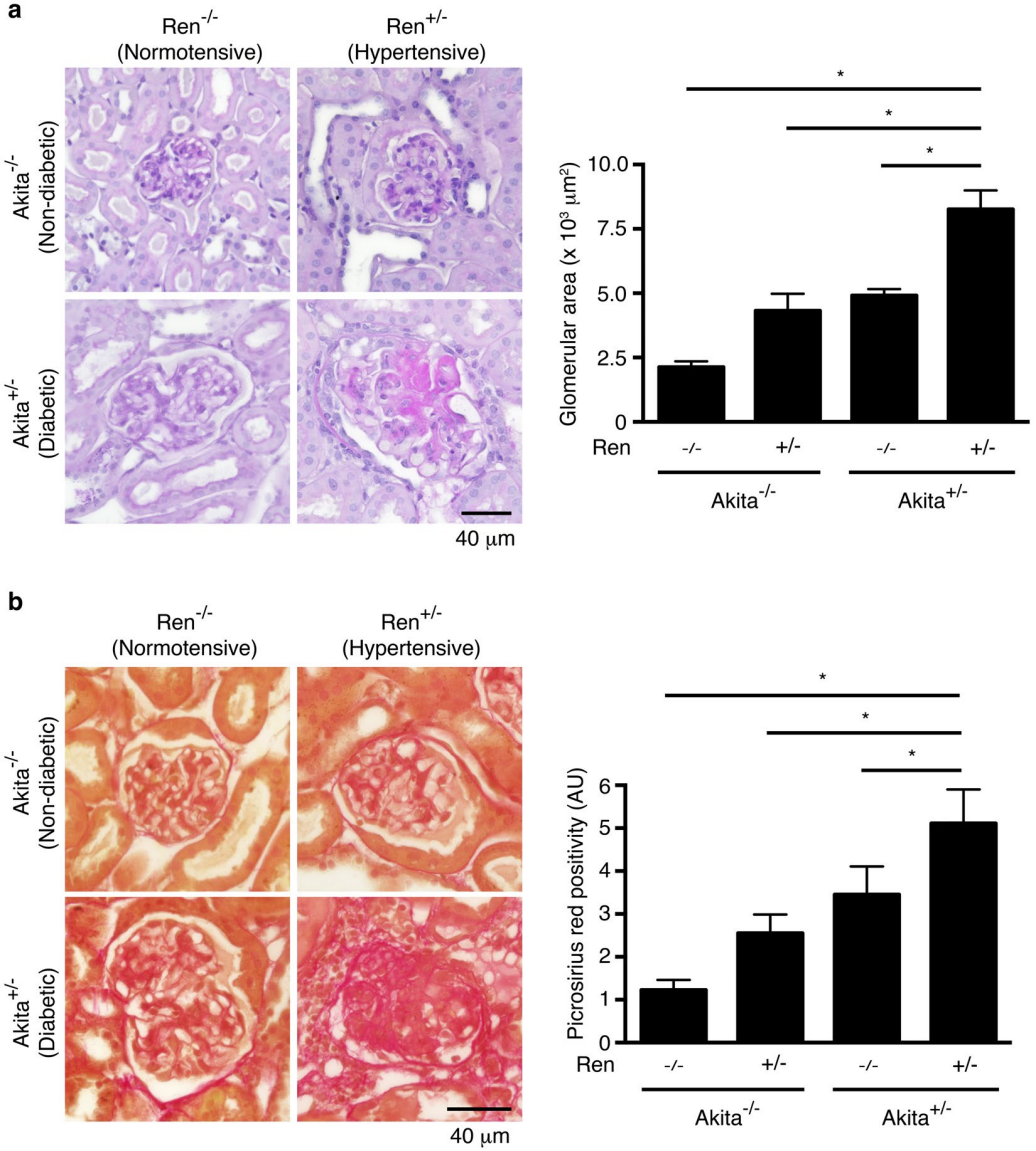
Akita^+/−^ Ren^+/−^ mice develop significant glomerular injury. (**a**) Kidney sections were stained with Periodic Acid-Schiff stain, and glomerular area was quantified. (**b**) Kidney sections were stained with picrosirius red to identify fibrillar collagen, and the degree of glomerulosclerosis was quantified. A one-way ANOVA with a post-hoc Fisher’s least significant difference analysis was performed. *p < 0.05. Scale bar: 40 μm. Abbreviations: AU, arbitrary units.

Kidneys and glomeruli from Akita^+/−^ Ren^+/−^ and Akita^+/−^ Ren^−/−^ mice were hypertrophied at study end (26 weeks of age, Table 1 and Fig. 2a). In addition, diabetic, hypertensive Akita^+/−^ Ren^+/−^ mice were noted to have advanced glomerulosclerosis with significantly expanded mesangial matrix, as compared to wild type Akita^−/−^ Ren^−/−^ controls, as well as diabetic, normotensive Akita^+/−^ Ren^−/−^ mice (Fig. 2a,b). Many glomeruli in Akita^+/−^ Ren^+/−^ mice were also noted to have diffuse and nodular glomerulosclerosis (Fig. 2a).

**Figure 3:**
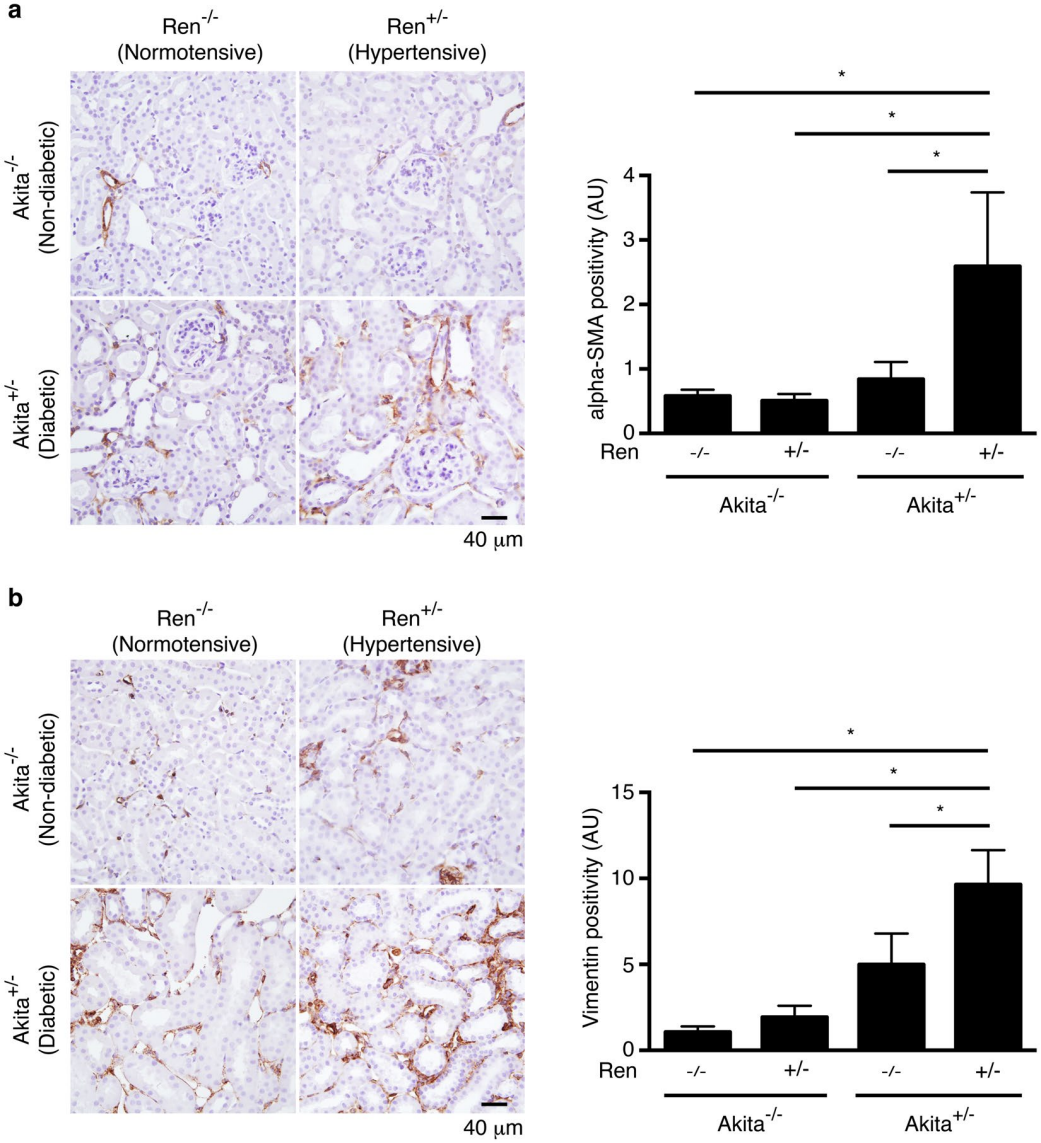
Interstitial myofibroblasts are increased in Akita^+/−^ Ren^+/−^ mice. Kidney sections were stained with antibodies specific either to: (**a**) alpha-smooth muscle actin (alpha-SMA) or (**b**) vimentin and digital quantification of antibody staining was performed. Representative images and quantification using both stains are shown. A one-way ANOVA with a post-hoc Fisher’s least significant difference analysis was performed. *p < 0.05. Scale bar: 40 μm. Abbreviations: AU, arbitrary units.

**Figure 4:**
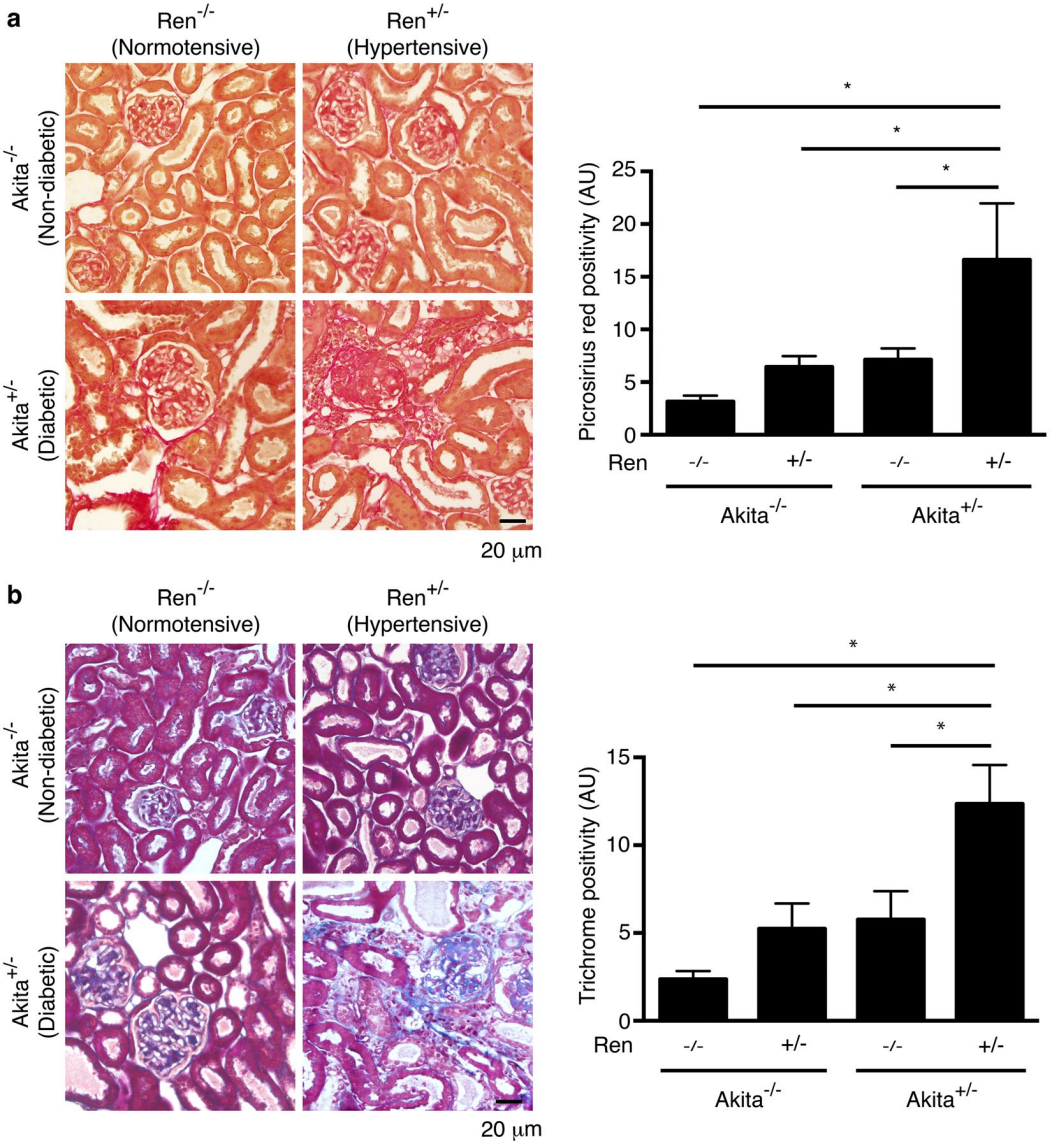
Akita^+/−^ Ren^+/−^ mice exhibit significant interstitial fibrosis. Kidney sections were stained with (**a**) picrosirius red to identify fibrillar collagen or (**b**) Masson’s Trichrome. Representative images and quantification of interstitial fibrosis using both stains are shown. A one-way ANOVA with a post-hoc Fisher’s least significant difference analysis was performed. *p < 0.05. Scale bar: 20 μm. Abbreviations: AU, arbitrary units.

**Figure 5:**
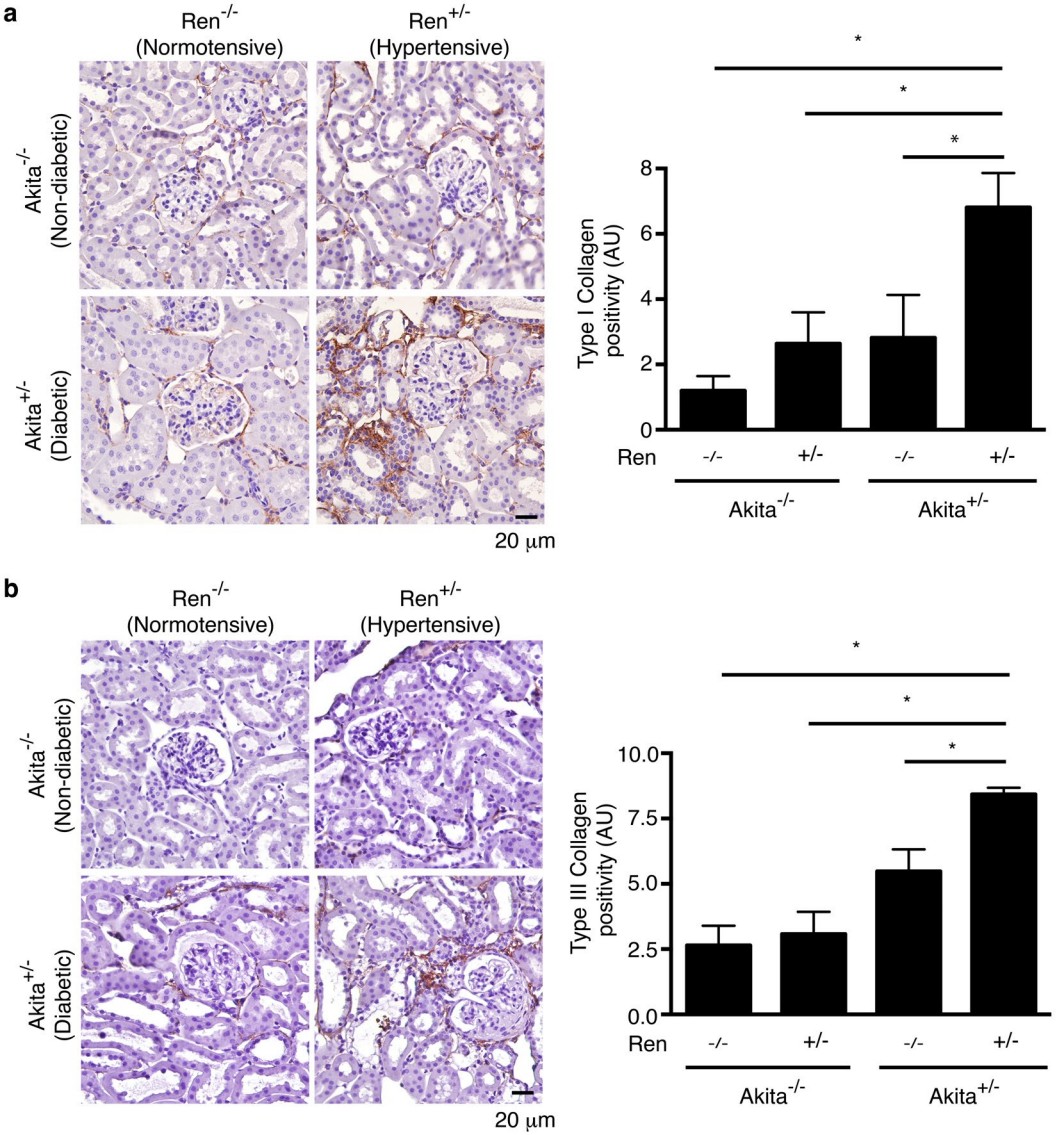
Collagen deposition is increased in Akita^+/−^ Ren^+/−^ mice. Kidney sections were stained with antibodies targeting (**a**) type I collagen or (**b**) type III collagen and digital quantification of antibody staining was performed. A one-way ANOVA with a post-hoc Fisher’s least significant difference analysis was performed. *p < 0.05. Scale bar: 20 μm. Abbreviations: AU, arbitrary units.

**Figure 6:**
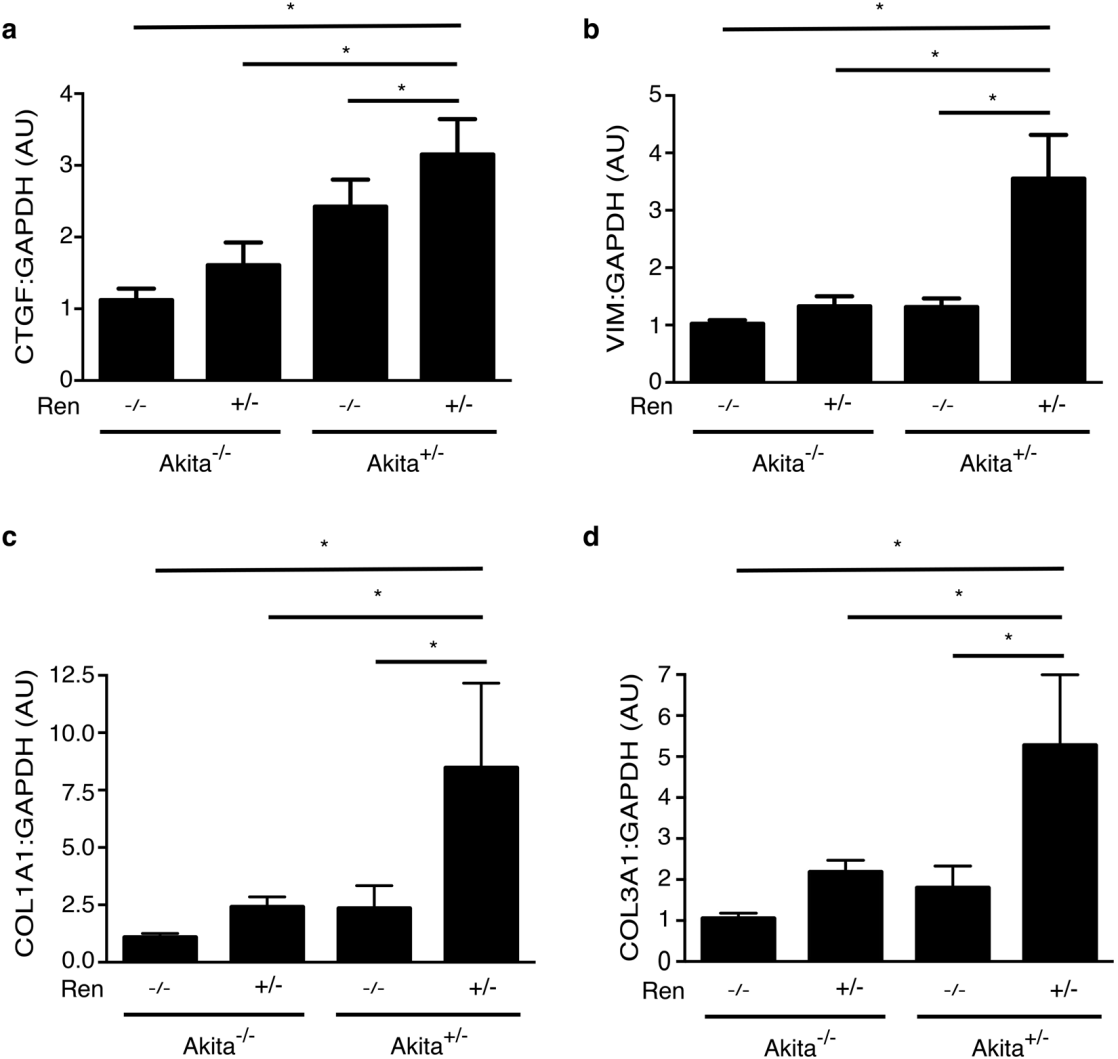
The expression of fibrosis-associated genes is increased in Akita^+/−^ Ren^+/−^ mice. (**a**) CTGF levels. (**b**) VIM (vimentin) levels. (**c**) COL1A1 levels. (**d**) COL3A1 levels. Levels of each transcript are presented relative to GAPDH. A one-way ANOVA with a post-hoc Fisher’s least significant difference analysis was performed. *p < 0.05. Abbreviations: AU, arbitrary units.

Of all the animal groups, only diabetic, hypertensive Akita^+/−^ Ren^+/−^ mice exhibited a significant interstitial fibrogenic response, as evidenced by increased staining for myofibroblast markers such as alpha-smooth muscle actin and vimentin (Fig. 3). Consistent with these findings, we also noted a significant increase in interstitial matrix staining only in Akita^+/−^ Ren^+/−^ mice (Figs 4 and 5). Semi-quantitative real time PCR corroborated these findings, with increases in fibrosis-associated genes noted most prominently in Akita^+/−^ Ren^+/−^ mice (Fig. 6).

### Increased YAP/TAZ protein levels and nuclear localization in Akita^+/−^ Ren^+/−^ mice

**Figure 7:**
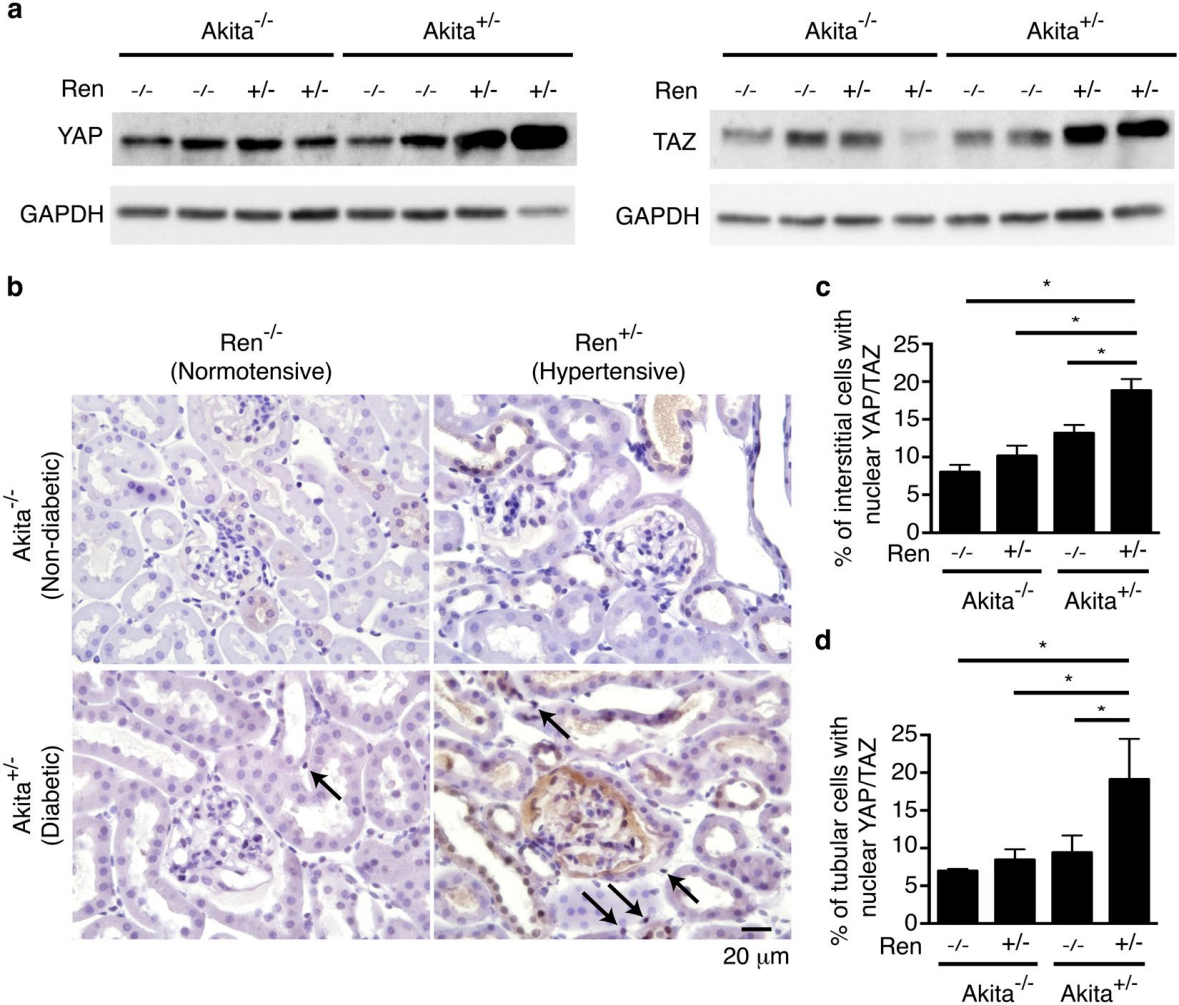
Akita^+/−^ Ren^+/−^ kidneys exhibit higher levels and nuclear localization of the pro-fibrotic proteins YAP and TAZ. (**a**) Equal amounts of kidney lysates (20 μg/lysate/membrane) were separated by SDS-PAGE and transferred on to two different membranes, with one membrane immunoblotted with an antibody directed against YAP (to detect total YAP), and a paired membrane immunoblotted with an antibody directed against YAP and TAZ (to detect total TAZ). Each of these membranes was then stripped and probed with an antibody directed against GAPDH. (**b**) Kidney sections were stained with an antibody targeting both YAP and TAZ, and counterstained with hematoxylin. Representative images are shown. The percentage of (**c**) interstitial cells and (**d**) tubular epithelial cells with primarily nuclear YAP/TAZ staining was quantified. Black arrows depict examples of interstitial cells with primarily nuclear YAP/TAZ staining. A one-way ANOVA with a post-hoc Fisher’s least significant difference analysis was performed. *p < 0.05. Scale bar: 20 μm.

We next examined the levels and subcellular localization of the pro-fibrotic transcription co-factors YAP and TAZ. Akita^+/−^ Ren^+/−^ mice demonstrated significantly increased YAP and TAZ protein levels, as compared to all other groups (Fig. 7a and Supplemental Fig. 2). Immunostaining demonstrated that both tubular epithelial and interstitial cells expressed increased levels of YAP and TAZ protein in Akita^+/−^ Ren^+/−^ mice, with localization primarily in the nuclei of these cells (Fig. 7b–d).

## Discussion

Interstitial fibrosis has long been recognized as a key driver of diabetic kidney disease progression, but our understanding of this process has been limited because most preclinical animal models fail to develop significant interstitial scarring. Here, we describe an easily generated mouse model of diabetic nephropathy that develops robust interstitial fibrosis and mimics many other features of advanced human diabetic kidney disease.

While historically much attention has focused on glomerular injury in diabetes, a strong body of evidence suggests that interstitial fibrosis is also a critical contributor to the pathogenesis of diabetic kidney disease. Multiple reports, for example, have linked interstitial fibrotic burden to both the degree and progression of renal dysfunction in patients with diabetes^4,25–27^. Interstitial matrix deposition is an important driving factor for diabetic kidney disease progression because it compromises tubular oxygen delivery by not only obliterating peritubular capillaries and thus diminishing tubulointerstitial microvascular blood flow, but also by lengthening the diffusion distance for oxygen between the remaining capillaries and tubular epithelial cells^5–7^. The resulting hypoxia triggers a cascade of events that can perpetuate renal injury, including inducing tubular cell apoptosis, inflammatory cell recruitment, and further fibroblast activation^5,6^. Interstitial scarring also changes the physical properties of the tubulointerstitium, as compliant cells are replaced with stiff extracellular matrix. This tissue stiffening can stimulate fibroblasts through the activation of pro-fibrotic mechanosensitive transcription factors such as YAP and TAZ^19,28–31^. Reflecting their increased interstitial fibrotic burden, Akita^+/−^ Ren^+/−^ mice demonstrated significant increases in interstitial nuclear YAP/TAZ staining, a marker of YAP/TAZ activation (Fig. 7).

YAP and TAZ are two related transcription co-factors that have recently been shown to be critical regulators of organ fibrosis, acting via both TGF-β-dependent and -independent pathways^19–22,32^. The activity of these factors is controlled primarily by the Hippo pathway^33,34^, composed of a core set of serine/threonine kinases that are regulated by a variety of biochemical^35–37^ and biomechanical stimuli^19,33,34,38,39^. Many of these stimuli are active in the diabetic kidney, such as angiotensin II^35,40^ and fibrosis-induced tissue stiffening^19,28^. Interestingly, a recent report demonstrated that in normotensive rodents with short term diabetes (2 weeks), only YAP levels are increased, while TAZ protein is reduced^41^. In contrast, we show, for the first time, that both YAP and TAZ appear to be increased and activated in the late stage, hypertensive, diabetic kidney, suggesting a role for these factors in driving diabetic renal fibrosis. It is thus possible that increased levels and activation of both YAP and TAZ occur only in later stage diabetic kidney disease, although future studies are needed to more definitively address this important question. Finally, we noted that YAP and TAZ localized to the nuclei of both interstitial and tubular epithelial cells in Akita^+/−^ Ren^+/−^ kidneys. As recent work has suggested that both YAP and TAZ can mediate pro-fibrotic effects in tubular epithelial cells^23,32^ and interstitial fibroblasts^21^ within the kidney, future studies will be required to tease out the individual contributions of YAP/TAZ activity in each of these cell types.

Over the years, many rodent models have been developed to study the pathogenesis of diabetic nephropathy. Most models develop manifestations of early human disease, such as hyperfiltration, albuminuria, mesangial matrix expansion, and glomerular basement membrane thickening^3,42^. Very few, however, develop key features of later stage disease, such as interstitial fibrosis, arteriolar hyalinosis, and reduced glomerular filtration rate^3,42^. Interstitial fibrosis has been a particularly difficult manifestation to replicate in rodents, with even preferred models of disease such as the streptozotocin (STZ)-administered eNOS knockout mouse^43,44^ and the db/db eNOS knockout mouse^45^ failing to develop significant interstitial scarring.

Renin angiotensin system (RAS) activation has long been recognized as a critical driver of diabetic nephropathy progression, with angiotensin II in particular having been shown to stimulate fibroblast matrix production both directly and indirectly through the release of transforming growth factor-β^46^. Consistent with its important role in diabetic nephropathy pathogenesis, inhibition of the renin-angiotensin system attenuates interstitial scar deposition^10,11^, and thus slows progression of renal dysfunction in the diabetic kidney^15,17^. Recognizing its importance as a pro-fibrotic stimulus, a number of groups have induced diabetes pharmacologically or genetically in rodent models that overexpress renin. Early experience was developed in the rat, with investigators first inducing diabetes via STZ administration in the (mREN-2)27 rat that overexpresses the mouse *Ren-2* gene^47^. The STZ-(mREN-2)27 rat develops significant hypertension and albuminuria, accompanied by an early increase in glomerular filtration followed by a subsequent decline that mimics the functional changes seen in human diabetic kidney disease^47^. Importantly, the STZ-(mREN-2)27 rat also develops profound glomerulosclerosis and interstitial fibrosis that, like human disease, is responsive to RAS inhibition^47^. More recently, Conway *et al*. described an inducible model of renin overexpression using rats with the mouse *Ren-2* gene under the control of the cytochrome P450a1 promoter. Feeding with indole-3-carbinol induced expression of the murine *Ren-2* gene, leading to moderate hypertension that, when combined with STZ-induced diabetes, resulted in significant interstitial fibrosis and other manifestations of diabetic nephropathy similar to those seen in the STZ-(mREN-2)27 rat^48^.

While helpful for the study of diabetes-induced renal fibrosis, these rat models do not permit the use of the many powerful genetic tools that have been developed in mice. Recognizing this, a number of groups have recently examined the effects of renin overexpression in diabetic mouse models. Harlan *et al*. infected various insulinopenic and insulin resistant murine models of diabetes with a renin-encoding adeno-associated virus (AAV) at 12 weeks of age, noting strain- and model-dependent variability in the degree of renal dysfunction and structural injury observed at 12 weeks following virus injection^49^. The model which developed the most severe disease was the AAV-infected uninephrectomized db/db mouse, which demonstrated increases in glomerulosclerosis and interstitial fibrosis when assessed by semi-quantitative measures, as well as glomerular filtration rate reduction at later disease stages. While promising, this model is resource-intensive, requiring both uninephrectomy and AAV injection^49^. Along similar lines, Thibodeau *et al*. induced diabetes in Ren^+/−^ mice, which overexpress human renin under the control of the constitutively active transthyretin promoter. Following induction of diabetes with either streptozotocin or intercrossing with OVE26 diabetic mice that develop diabetes neonatally due to β-cell destruction^50^, Ren^+/−^ mice developed significant hypertension, albuminuria, reduced glomerular filtration, and marked glomerulosclerosis^51^. Interstitial fibrosis was not examined quantitatively in these models, and so detailed assessment of fibrotic burden was not performed. Trichrome-stained images of the OVE26-Ren^+/−^ kidneys did suggest the development of some interstitial disease, although the exact amount was not quantified. In contrast, STZ-Ren^+/−^ mice were noted to have a much milder phenotype^51^.

The development of these murine models clearly represents a major advance, although to date an in-depth analysis of their interstitial fibrotic burden has yet to be performed. In the current study, we demonstrate that male Ren^+/−^ mice on a FVB/n background crossed with Akita^+/−^ mice on a C57BL/6 background develop many features of advanced human diabetic kidney disease, including hypertension, reduced glomerular filtration rate, albuminuria, and glomerulosclerosis. Importantly, using a wide array of techniques, we further demonstrate that these Akita^+/−^ Ren^+/−^ mice exhibit a consistent and robust increase in interstitial fibrosis (Figs 2–6). To our knowledge, we are the first to demonstrate and quantify this interstitial fibrotic phenotype in an easily generated mouse model of diabetes.

Our Akita^+/−^ Ren^+/−^ mouse model has a number of limitations. Firstly, due to its constitutive overexpression of human pro-renin, the Ren^+/−^ mouse develops hypertension at an early age, unlike most humans with diabetic kidney disease, in whom hypertension usually develops later in life. Secondly, like all insulinopenic models of diabetes, the Akita^+/−^ Ren^+/−^ mouse does not reflect the disturbed metabolic phenotype of humans with type 2 diabetes. It is likely that these and other variations from the human setting result in some differences in structural and functional manifestations of diabetic kidney injury. Finally, while exhibiting a significant increase in serum creatinine levels compared with non-diabetic mice, the Akita^+/−^ Ren^+/−^ mouse, at least by 26 weeks of age, develops only a mild reduction in glomerular filtration rate when compared with its standard normotensive Akita^+/−^ Ren^−/−^ counterpart (Table 1). It is possible that, with longer follow-up, the more severe fibrotic injury seen in Akita^+/−^ Ren^+/−^ mice will lead to a greater reduction in glomerular filtration rate, although future studies will be required to address this question. Nonetheless, our data clearly demonstrate that the Akita^+/−^ Ren^+/−^ mouse develops a robust interstitial fibrotic phenotype by 26 weeks of age, along with many other structural and functional features that mirror advanced human diabetic kidney disease.

Here we describe a new, easily generated mouse model of advanced human diabetic kidney disease that, unlike most other mouse models, develops significant interstitial fibrosis and reduced glomerular filtration rate. As interstitial fibrosis is a key driver of diabetic nephropathy progression, the Akita^+/−^ Ren^+/−^ mouse should provide new insights into disease pathogenesis, and also a means to test novel anti-fibrotic agents in the diabetic kidney.

## Methods

### Animals

All animal studies were approved by the St. Michael’s Hospital Animal Care Committee, and were conducted in accordance with Canadian Council on Animal Care guidelines. Mice were housed in a temperature-controlled environment with a 12 hour light:12 hour dark cycle, and provided free access to standard mouse chow and water. Ren^+/−^ mice on a FVB/n background expressing a single copy of a modified human pro-renin transgene under the control of a truncated mouse transthyretin promoter were a kind gift of Dr. Christopher Kennedy^51^. Female Ren^+/−^ mice were bred against male C57BL/6 Ins2^Akita^ (Akita^+/−^, Jackson Laboratory, Bar Harbor, ME) mice to generate Akita^+/−^ Ren^+/−^ (n = 4), Akita^+/−^ Ren^−/−^ (n = 6), Akita^−/−^ Ren^+/−^ (n = 10), and Akita^−/−^ Ren^−/−^ (n = 16) littermates. Only male mice were used, as female Akita^+/−^ mice do not consistently develop diabetes. Study mice were followed for a total of 26 weeks. Capillary blood glucose levels were tested after a 4 hour fast, and systolic blood pressure was measured monthly in anesthetized mice (1% isoflurane) using a CODA noninvasive tail cuff system (Kent Scientific, Torrington, CT). Prior to sacrifice, mice underwent 24 hour metabolic caging, and urinary albumin excretion was determined using an AssayMax mouse albumin ELISA kit (Assaypro, St. Charles, MO). Blood was collected by aortic puncture and blood hemoglobin A_1c_ levels were measured using an A1CNow + kit (Polymer Technology Systems, Indianapolis, IN). Serum and urine creatinine levels were measured using a picric acid based method. At study end (26 weeks of age), a surgical plane of anesthesia was induced with 5% isoflurane, and then mice were sacrificed by cervical dislocation.

### Histology

At study end, the right kidney was excised and stored in RNAlater (Invitrogen, Cat. #AM7024) or snap-frozen in liquid nitrogen. The animal was then perfusion-exsanguinated and whole body fixation achieved with perfusion of 10% neutral buffered formalin at 20 mL/min. The length of the left tibia was also recorded.

Following perfusion-exsanguination, the left kidney was excised, paraffin-embedded, and sectioned. Kidney sections were stained with Periodic Acid-Schiff stain (PAS, MilliporeSigma, Cat. #: 395B-1KT), Picrosirius Red (MilliporeSigma, Cat. #365548), Masson’s Trichrome (MilliporeSigma, Cat. #: HT15-1KT), or antibodies directed against alpha-smooth muscle actin (1:100 dilution, Agilent Dako, Santa Clara, CA, Cat. #M0851), type I collagen (1:200 dilution, Southern Biotech, Birmingham, AB, Cat. #1310-01)^52^, type III collagen (1:200 dilution, Southern Biotech, Cat. #1330-01)^53^, vimentin (1:500 dilution, Abcam, Cambridge, UK, Cat. #92547), Wilms Tumor 1 (WT1, 1:2000 dilution, Abcam, Cat. #89901)^54^, or YAP/TAZ (1:100 dilution, Cell Signaling Technology, Danvers, MA, Cat. #8418)^19^. Primary antibodies were detected with the following horseradish peroxidase-conjugated secondary antibodies: donkey anti-goat IgG (1:500 dilution, Santa Cruz Biotechnology, Dallas, TX, Cat. #sc-2020), donkey anti-mouse IgG (1:2000 dilution, ImmunoResearch, West Grove, PA, Cat. #715-035-150), and goat anti-rabbit IgG (1:100 dilution, Santa Cruz Biotechnology, Cat. #sc-2030). For assessment of glomerular area, a minimum of 20 glomeruli cut through the vascular hilum were identified per kidney using Periodic Acid-Schiff stained sections, and glomerular area quantified using ImageJ (NIH, Bethesda, MD). For assessment of glomerulosclerosis, a minimum of 20 glomeruli were identified per kidney using picrosirius red-stained kidney sections, and the images analyzed in a blinded fashion using Aperio Imagescope software. For assessment of cortical fibrosis and myofibroblast marker expression, five 20X random, non-overlapping images per kidney were taken with an Olympus Upright BX50 Microscope, and the images analyzed in a blinded fashion using Aperio Imagescope software as previously described^55^. In the case of YAP/TAZ staining, the percentage of interstitial cells with primarily nuclear staining was manually determined in a blinded fashion from the five collected 20X images. For podocyte quantification, WT1^+^ cells were manually counted in a blinded fashion from a minimum of 30 randomly selected glomeruli per kidney section, and presented as a percentage of the total number of cells in each glomerulus. The average percentage of glomerular cells that were WT1^+^ was then calculated for each kidney section.

### Quantitative real time PCR (RT-PCR)

Following tissue homogenization, RNA extraction, and reverse transcription, quantitative RT-PCR was performed using SYBR green on an ABI Prism 7900HT Fast PCR System (Applied Biosystems, Foster City, CA) to analyze the differences in the mRNA levels of COL1A1, COL3A1, CTGF, VIM (vimentin), and GAPDH. Primer sequences (IDT Corp., Toronto, ON, Canada) were as follows: COL1A1 forward: GAG AAC CAG CAG AGC CA, COL1A1 reverse: GAA CAA GGT GAC AGA GGC ATA; COL3A1 forward: CAT TGC GTC CAT CAA AGC C, COL3A1 reverse: GAA AGG ATG GAG AGT CAG GAA; CTGF forward: CCC TAG CTG CCT ACC GAC T, CTGF reverse: GGT AAC TCG GGT GGA GAT GC; VIM forward: CGG AAA GTG GAA TCC TTG CA, VIM reverse: CAC ATC GAT CTG GAC ATG CTG T; GAPDH forward: AAT GGT GAA GGT CGG TGT T; GAPDH reverse: GTG GAG TCA TAC TGG AAC ATG TAG.

### Protein isolation and immunoblotting

Kidneys were dissected manually and whole cell lysates were prepared using lysis buffer containing cocktail inhibitors (cOmplete Mini, EDTA-free, Roche, Germany). The lysates were separated by SDS-PAGE and transferred on to PVDF membranes. Immunoblotting was performed using the following primary antibodies raised in rabbit against: YAP/TAZ (1:1000 dilution, Cell Signaling Technology, Cat. #8418S)^19^, YAP (1:1000 dilution, Cell Signaling Technology, Cat. #14074S)^56^, and GAPDH (1:10,000 dilution, Cell Signaling Technology, Cat. #2118S). Primary antibodies were detected with horseradish peroxidase-conjugated donkey anti-rabbit IgG (1:10,000 dilution, Santa Cruz Biotechnology, Cat. #sc-2030).

### Statistics

Data presented are mean ± standard error of the mean (SEM). Between-group differences were measured using one-way ANOVA with Fisher’s least significant difference post-hoc analysis where appropriate. Statistical analysis was performed using Graphpad Prism for Mac 6.0 (Graphpad Software, San Diego, CA). A p value < 0.05 was considered significant.

### Study approval

All animal studies were approved by the St. Michael’s Hospital Animal Ethics Committee, and conformed to the Canadian Council on Animal Care guidelines.

## Supplementary information

Supplemental Information

## Data Availability

All data generated or analyzed during this study are including in this published article.
