# Supplementary material for: A new, easily generated mouse model of diabetic kidney fibrosis
Source: Sci Rep. 2019 Aug 29;9:12549. doi: 10.1038/s41598-019-49012-4 (PMC6715679; doi:10.1038/s41598-019-49012-4)
Supplement: Supplementary file 1 — Supplemental Information [file 41598_2019_49012_MOESM1_ESM.docx]

**A new, easily generated mouse model of diabetic kidney fibrosis**

Xiaolin He^1^, Tianzhou Zhang^1^, Monica Tolosa^1^, Santosh Kumar Goru^1^, Xiaolan Chen^1,2^, Paraish S. Misra^1^, Lisa A. Robinson^3^, Darren A. Yuen^1,*^

^1^ Keenan Research Centre for Biomedical Science, Li Ka Shing Knowledge Institute, St. Michael’s Hospital, Toronto, Ontario, Canada.

^2^ Department of Respiratory and Critical Care Medicine, Beijing Shijitan Hospital, Capital Medical University, Beijing, People’s Republic of China.

^3^ Hospital for Sick Children, Toronto, Ontario, Canada.

Running title: A new model of diabetic kidney fibrosis

**SUPPLEMENTAL INFORMATION**

**Supplemental Figure 1**

**
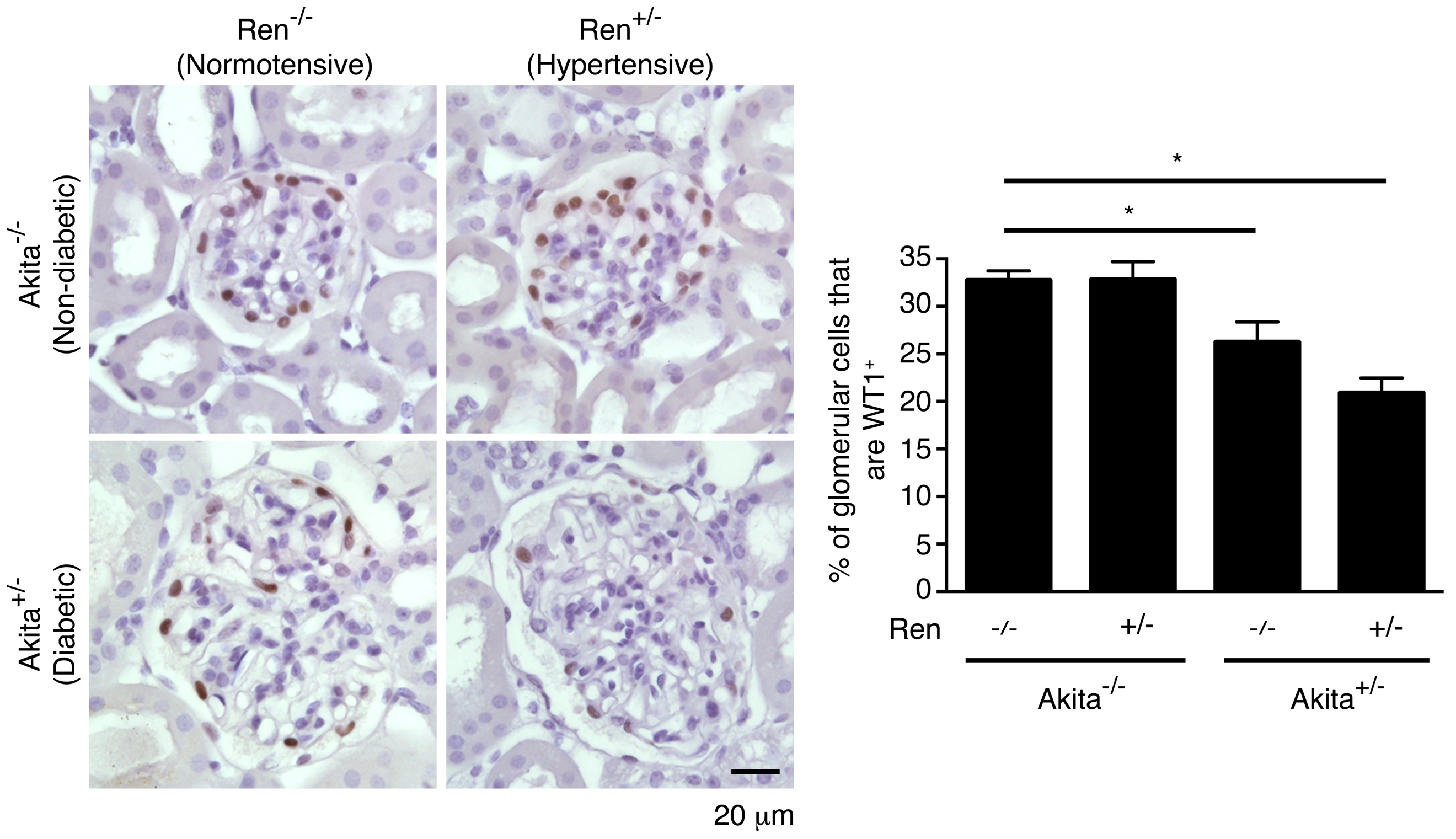
**

**Supplemental Figure 1: Podocyte number is reduced in Akita^+/-^ Ren^+/-^ mice.**

Kidney sections were stained with an antibody directed against WT1, a podocyte-enriched antigen. Representative images are shown, as well as the results of digital quantification of antibody staining. A one-way ANOVA with a post-hoc Fisher’s least significant difference analysis was performed. * p < 0.05. Scale bar: 20 μm.

**Supplemental Figure 2**

**
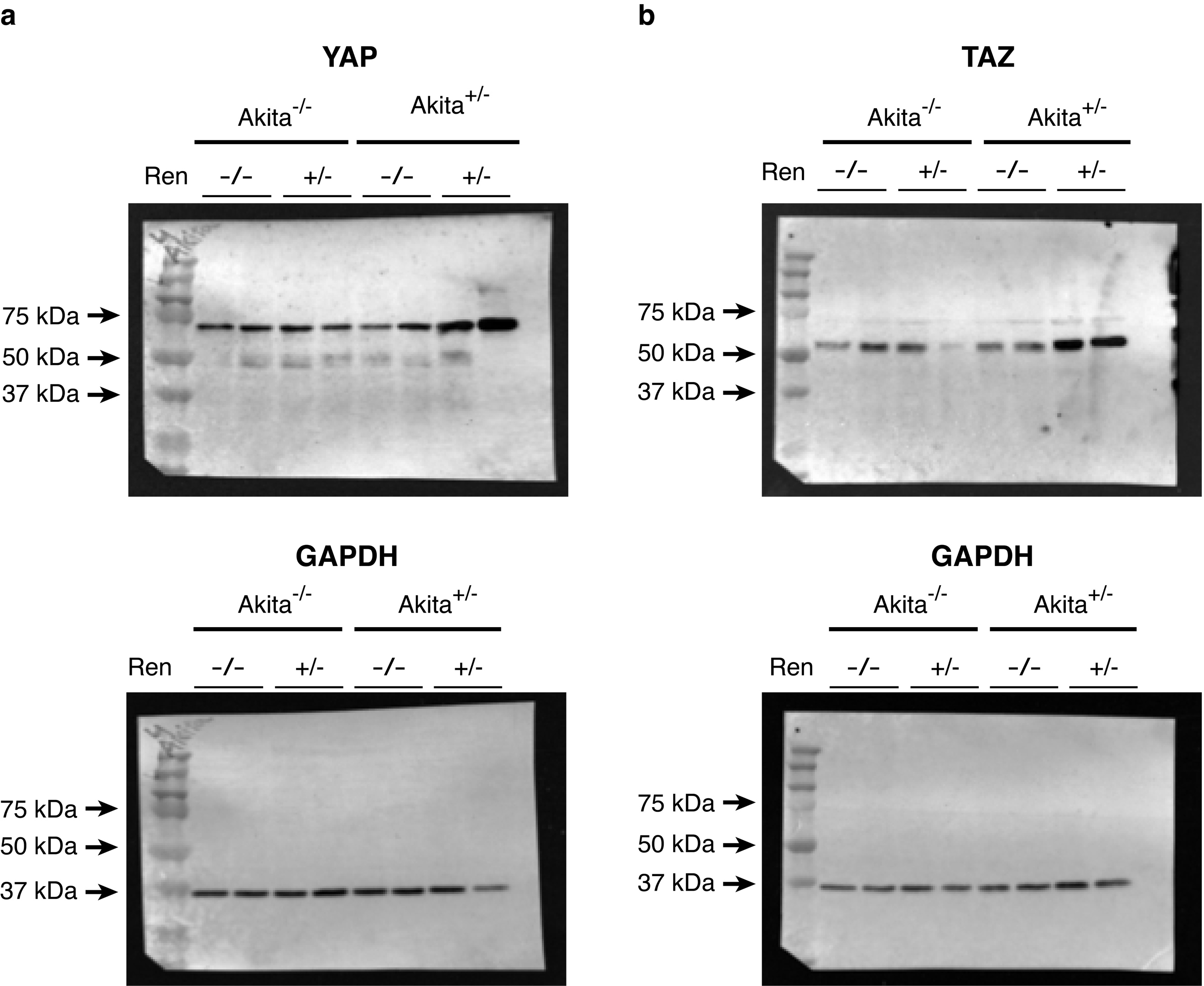
**

**Supplemental Figure 2: Akita^+/-^ Ren^+/-^ kidneys exhibit higher levels of YAP and TAZ (raw blots).**

Equal amounts of kidney lysates (20 μg/lysate/membrane) were separated by SDS-PAGE and transferred on to two different membranes, with (a) one membrane immunoblotted with an antibody directed against YAP (to detect total YAP, top panel), and (b) a paired membrane immunoblotted with an antibody directed against YAP and TAZ (to detect total TAZ, top panel). Each of these membranes was then stripped and probed with an antibody directed against GAPDH (bottom panels).
